# Consistent Errors in First Strand cDNA Due to Random Hexamer Mispriming

**DOI:** 10.1371/journal.pone.0085583

**Published:** 2013-12-30

**Authors:** Thomas P. van Gurp, Lauren M. McIntyre, Koen J. F. Verhoeven

**Affiliations:** 1 Netherlands Institute of Ecology (NIOO-KNAW), Department of Terrestrial Ecology, Wageningen, The Netherlands; 2 Genetics Institute, University of Florida, Gainesville, Florida, United States of America; University of North Carolina at Charlotte, United States of America

## Abstract

Priming of random hexamers in cDNA synthesis is known to show sequence bias, but in addition it has been suggested recently that mismatches in random hexamer priming could be a cause of mismatches between the original RNA fragment and observed sequence reads. To explore random hexamer mispriming as a potential source of these errors, we analyzed two independently generated RNA-seq datasets of synthetic ERCC spikes for which the reference is known. First strand cDNA synthesized by random hexamer priming on RNA showed consistent position and nucleotide-specific mismatch errors in the first seven nucleotides. The mismatch errors found in both datasets are consistent in distribution and thermodynamically stable mismatches are more common. This strongly indicates that RNA-DNA mispriming of specific random hexamers causes these errors. Due to their consistency and specificity, mispriming errors can have profound implications for downstream applications if not dealt with properly.

## Introduction

RNA-seq is a widely used tool for transcriptome analysis and gene expression estimation. Most commonly, mRNA is fragmented followed by reverse transcription into first strand cDNA primed by random hexamers. Subsequently, second strand cDNA is synthesized from first strand cDNA by DNA polymerase, again initiated by random hexamer priming. Bias in hexamer distribution and CG content affects abundance estimates and several bias correction algorithms have been developed e.g. [1-5]. 

Apart from bias in hexamer priming sites, random hexamer mispriming has recently been implicated in sequence read to reference mismatches. Because mismatches mainly occur in the first seven nucleotides of first strand cDNA [4,6] and are observed in transcriptomic but not genomic sequencing datasets [1], RNA-DNA mispriming of random hexamers during first strand cDNA synthesis has been suggested as a likely explanation for the observed sequence mismatches [4,6,7]. It is important to recognize such technical artifacts because they might obviate biological interpretation of observed SNP patterns or RNA-editing [6-9].

This paper deals with sequence read to reference mismatches commonly observed in RNA-sequencing data. Mismatches are defined as any position in sequencing reads that deviate from the reference to which these reads align. Mismatches can reflect 1) true biological variation, both in genomic DNA or caused by RNA-editing, or 2) errors in the library preparation process caused by hexamer mispriming or PCR errors, and finally 3) sequencing errors caused by the erroneous identification of bases in the sequencing process. We focus on errors that most likely arise in the library preparation phase. During RNA-seq library preparation, polyA+ RNA is fragmented and reverse transcribed into first strand cDNA initiated by random hexamer priming. We refer to mismatches between the reference fragment and the observed sequence that are caused by RNA-DNA hexamer mispriming during first strand cDNA synthesis as RD-mismatches. Similarly, we use the term DD-mismatches to describe errors caused by DNA-DNA hexamer mispriming during second strand cDNA synthesis.

Errors caused by random hexamer mispriming have thus far received limited attention. While read trimming to exclude error-rich first stretches of RNA-seq reads is done in some studies [10,11], there is currently limited insight into the problem and no general consensus exists on how to efficiently deal with it in RNA-seq data. Here we perform a detailed analysis of both RD-mispriming and DD-mispriming in RNA-seq data in order to further our understanding of the causes and possible consequences of errors associated with random hexamer binding.

## Materials and Methods

We conducted a RNA-seq experiment of *Taraxacum officinale* RNA mixed with ERCC RNA spikes. Twenty-three libraries were multiplexed using Illumina’s multiplex sequencing assay and pooled with a 2% ERCC spike [12]. Subsequently they were sequenced on 2 Hiseq lanes yielding a total of 2.4 million 100bp read pairs mapping to the ERCC spike set. Adapter trimming and quality filtering was done using Fastq-mcf v1.0.3-r152 with the following settings (-x 0 –k 0 –q 20). Mapping was done with BWA 0.6.1-r104 with default settings. Unpaired reads or reads from pairs with incorrect insert sizes containing adapter remnants at the 3’ end were excluded from this analysis. A custom python script (available at http://goo.gl/5c9DaZ) was used to identify positional errors in forward and reverse mapping reads from the bam file, data are deposited in the SRA with reference number SRR954526. ERCC RNA-seq data described in [4]; GSM517062) were mapped and analyzed with the same settings.

## Results and Discussion

**Figure 1:**
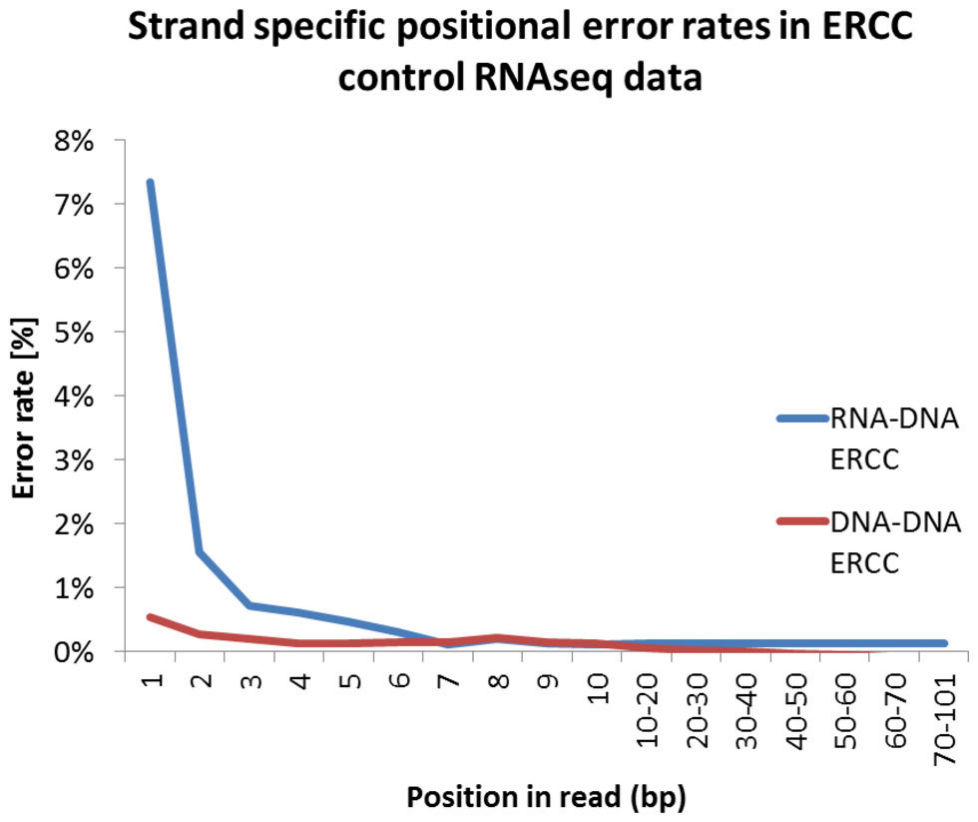
Read position effect on sequence mismatches. Sequence mismatch rates in first (RNA-DNA) and second (DNA-DNA) strand cDNA of reads mapping to ERCC sequences.

We analyzed two independently generated RNA-seq datasets, focusing on reads mapping to ERCC spikes which are artificial RNA fragments of known sequence that are added during library preparation[4]. Reverse mapping reads represent first strand cDNA and forward mapping reads represent second strand cDNA. Because the reference strand is known for all ERCC’s, deviations represent library preparation or sequencing errors, not true biological variation. For all positions in reverse and forward reads, substitution errors were calculated by parsing the sequence and MD tag of all reads in the SAM file. Here, the mismatching and expected nucleotide as well as position is denoted. Consistent with previous results [4,6,8], we observed that per position nucleotide mismatch rates are higher for the first seven nucleotides compared to the rest of the sequence reads. As we can separate first and second strand synthesis we show that first strand synthesis mismatch rate is higher, consistent with RNA-DNA hexamer mispriming (Figure 1). Of importance, these are bases called with high quality, and thus do not likely represent sequencing error. A slight increase in mismatch rate was also detected for initial nucleotides of second strand cDNA, suggesting that DNA-DNA hexamer binding is not insensitive to mispriming errors. Sequence to reference mismatches are not limited to the first nucleotide as commonly observed in Illumina sequencing data [13], but similar to first strand cDNA, show higher rates in the first seven nucleotides compared to the rest of the read.

**Figure 2:**
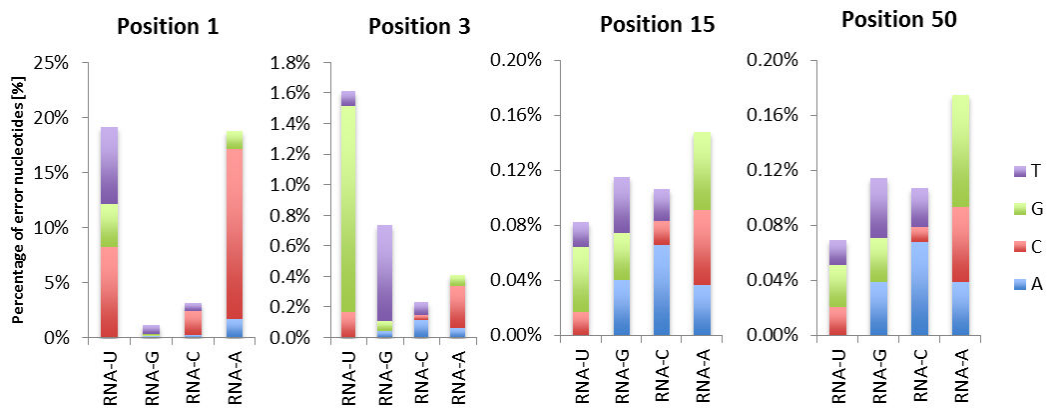
The mismatch rate and distribution for the first, third, 15th and 50th positions of first strand cDNA. For all 4 nucleotides present in RNA the distribution of mismatching nucleotides is shown at selected positions. Mismatch rates are highest for first strand cDNA reads starting with T or A. For position three mismatches are mostly due to RNA-U vs DNA-G mispriming. Per nucleotide mismatch distributions are highly variable for the first seven positions, whereas they are consistent from position 7 onwards.

**Figure 3:**
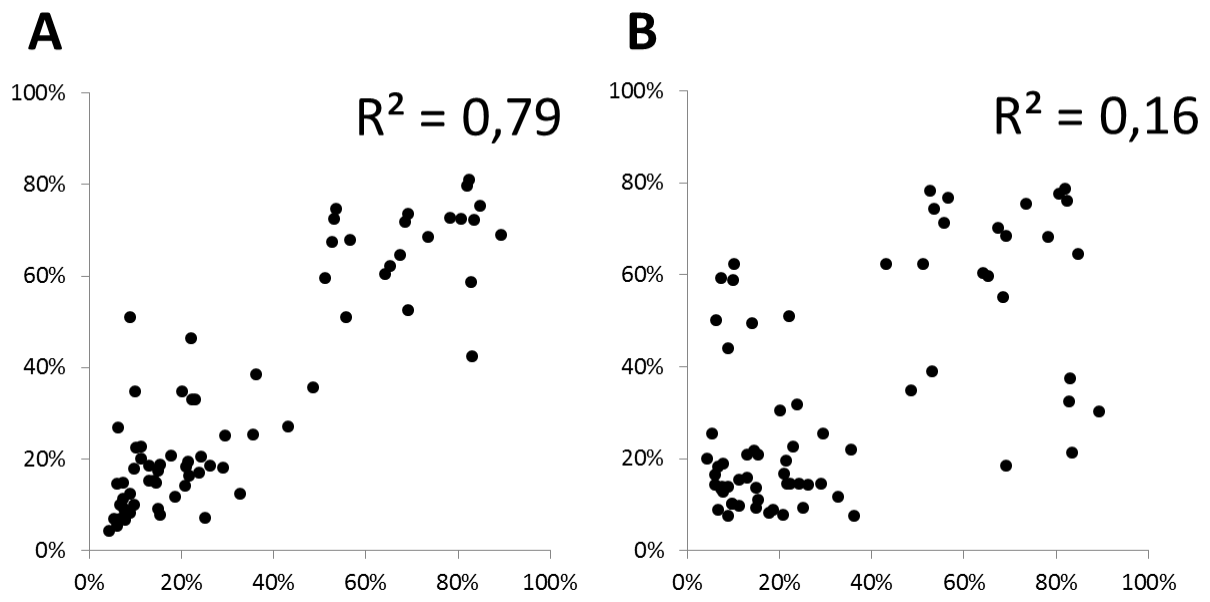
Mismatch error pattern correlation between two independent datasets. Mismatch error patterns observed in ERCC spike reads are correlated between independent data sets for read position 1-6 (panel A) but not for subsequent positions (position 7-20, panel B). For each position the distribution of errors over the 12 mispriming possibilites was determined (4 nucleotides x 3 mispriming options, summing to 100% per position) and plotted between the dandelion RNA-seq ERCC-dataset (see Figure 1) and ERCC RNA-seq data described in Jiang (2011; GSM517062). The correlation in error distributions between the two data sets shows consistency of specific mismatching errors only in the hexamer binding region (panel A).

First strand cDNA mismatches in the first seven base pairs corresponding to the hexamer binding site and the base immediately downstream of this show position-dependent and nucleotide-dependent mismatch patterns (Figure 2). These specific mismatch patterns differ markedly from the mismatch rates and distribution observed downstream of base seven caused by sequencing or PCR errors (Figure 3). RNA-seq reads derived from first strand cDNA that that start with A or T misprime in 20% of the cases, in which rA-dC and rU-dC mispriming are most common. In positions 2-6 ~65% of mispriming events consist of rU-dG and rG-dT, which are most stable among all 12 possible RNA-DNA misprimed pairs [14]. Overall, hexamer mispriming occurs most commonly at RNA binding sites with uracil, whereas cytosine in RNA prevents most hexamers from mispriming (Figure 2). 

Thus we conclude that mispriming is non-random and can be heavily biased, as RNA binding sites with a U at positions three and four (relative to the hexamers 5’ end) misprime with hexamers having a G at that position in ~88% of the mispriming cases. These specific mismatch patterns were observed consistently in two independent data sets (Figure 3). The distribution, type and repeatability of mismatch patterns demonstrate that not all mispriming events have the same likelihood and RNA-DNA hexamer mispriming is the main source of error in the first seven nucleotides.

Consistent mismatch patterns observed in the first seven nucleotides of first strand cDNA will affect downstream applications such as de novo assembly, SNP calling and RNA-editing analysis. For instance, consistent and high (20%) mismatch rates can be problematic for k-mer assembly strategies, as these erroneous k-mers cannot be effectively combined with “true” k-mers. Because mispriming rates are not random and for some positions heavily biased (Figure 2) they can contribute to false positive variants that might not be detected based on filtering criteria such as read count or quality thresholds. Indeed, mismatches between human RNA-seq reads and the human reference genome have been interpreted as evidence for widespread RNA-editing [9]. Several reports highlight the overrepresentation of mismatches in the first six positions of first strand cDNA[6-8], suggesting hexamer mispriming on RNA as an explanation for the observed mismatches. Our study confirms the generality and specificity of hexamer mispriming, thus providing further support to this explanation. In fact, there are examples of filtering and preprocessing steps that counter the effect of random hexamer mispriming induced mismatches, however, their origin is not described. For example, empirically derived filtering parameters for putative somatic mutations used in Varscan 2 exclude the first ten bases of reads [15], and 5’ trimming has been applied to Illumina RNA-seq reads showing an increased N50, average and maximum contig length [10]. 

Identification of the hexamers that are most commonly involved in mispriming as well as the type and position of the mismatches this generates can aid in the design of strategies to counter the effects that these errors can have in downstream applications. Our results suggest that it could be useful to explore less aggressive approaches than trimming. Such approaches could include post-mapping correction of 5’ mismatches, modifications to random hexamer design to exclude commonly mispriming hexamers and specific bias correction models that mask or remove the observed mismatches in the first seven bases of reads. 

## Conclusion

Our analyses shows strong and consistent bias in sequence errors in 5’ ends of RNA-seq reads, which (1) strongly supports the hypothesis that random hexamer mispriming during first strand cDNA synthesis causes the errors, and (2) highlights the risk of errors in downstream applications as well as suboptimal data use. We conclude that technical artifacts in sequencing data are insufficiently described. Further research on random hexamer mispriming will inform optimized strategies to mitigate their negative effect on downstream analysis.
